# Neurogenesis and neuronal differentiation in the postnatal frontal cortex in Down syndrome

**DOI:** 10.1186/s40478-022-01385-w

**Published:** 2022-06-08

**Authors:** Emma C. Utagawa, David G. Moreno, Kristian T. Schafernak, Nicoleta C. Arva, Michael H. Malek-Ahmadi, Elliott J. Mufson, Sylvia E. Perez

**Affiliations:** 1 Department of Translational Neuroscience, Barrow Neurological Institute, 350 W Thomas Rd, Phoenix, AZ 85013 USA; 2 Department of Pathology and Laboratory Medicine, Phoenix Children’s Hospital, 1919 E Thomas Rd, Phoenix, AZ 85016 USA; 3 Department of Pathology and Laboratory Medicine, Ann and Robert H. Lurie Children’s Hospital of Chicago, 225 E Chicago Ave, Chicago, IL 60611 USA; 4 Banner Alzheimer’s Institute, 901 E Willetta St, Phoenix, AZ 85006 USA

**Keywords:** Down syndrome, Frontal cortex, Postnatal development, Calcium binding proteins, Neuronal maturation

## Abstract

Although Down syndrome (DS), the most common developmental genetic cause of intellectual disability, displays proliferation and migration deficits in the prenatal frontal cortex (FC), a knowledge gap exists on the effects of trisomy 21 upon postnatal cortical development. Here, we examined cortical neurogenesis and differentiation in the FC supragranular (SG, II/III) and infragranular (IG, V/VI) layers applying antibodies to doublecortin (DCX), non-phosphorylated heavy-molecular neurofilament protein (NHF, SMI-32), calbindin D-28K (Calb), calretinin (Calr), and parvalbumin (Parv), as well as β-amyloid (APP/Aβ and Aβ_1–42_) and phospho-tau (CP13 and PHF-1) in autopsy tissue from age-matched DS and neurotypical (NTD) subjects ranging from 28-weeks (wk)-gestation to 3 years of age. Thionin, which stains Nissl substance, revealed disorganized cortical cellular lamination including a delayed appearance of pyramidal cells until 44 wk of age in DS compared to 28 wk in NTD. SG and IG DCX-immunoreactive (-ir) cells were only visualized in the youngest cases until 83 wk in NTD and 57 wk DS. Strong SMI-32 immunoreactivity was observed in layers III and V pyramidal cells in the oldest NTD and DS cases with few appearing as early as 28 wk of age in layer V in NTD. Small Calb-ir interneurons were seen in younger NTD and DS cases compared to Calb-ir pyramidal cells in older subjects. Overall, a greater number of Calb-ir cells were detected in NTD, however, the number of Calr-ir cells were comparable between groups. Diffuse APP/Aβ immunoreactivity was found at all ages in both groups. Few young cases from both groups presented non-neuronal granular CP13 immunoreactivity in layer I. Stronger correlations between brain weight, age, thionin, DCX, and SMI-32 counts were found in NTD. These findings suggest that trisomy 21 affects postnatal FC lamination, neuronal migration/neurogenesis and differentiation of projection neurons and interneurons that likely contribute to cognitive impairment in DS.

## Introduction

Down syndrome (DS) is a genetic disorder caused by trisomy of chromosome 21 that is characterized by developmental delay, intellectual disability and memory impairment linked, in part, to a reduction in the volume of the frontal cortex (FC), hippocampus and cerebellum [2, 25, 62]. By middle age, individuals with DS display beta-amyloid (Aβ) plaques and tau-laden neurofibrillary tangles (NFTs) and are at a greater risk of developing AD-type dementia [33, 48]. Children with DS display deficits in cognitive function, attention, emotional behavior, executive function, working memory and language, in part associated with damage to the six-layered FC [22, 91]. In neurotypical development, cortical laminar differentiation begins between 26 and 29 wk of gestation including a rapid appearance of pyramidal neurons and interneurons in layers III and V, resulting in a mature six-layered cortex at birth [61]. Postnatal development also plays a critical role in the continued maturation of a well-differentiated FC, which involves tightly regulated spatiotemporal processes mediating cellular proliferation, migration, targeting and connectivity, and perturbations of these events contribute to intellectual disability [19, 78].

Fetal and neonatal DS brains show decreases in cell number, disorganized and delayed cortical lamination, and abnormalities in synaptodentritic processes [28, 32, 42, 72, 86]. Moreover, prior to 24 wk of gestation there are reductions in both cellular proliferation and radial glia in the DS neocortex [5, 14, 42, 44, 49]. These alterations have been associated with impaired neurogenesis during fetal gestation [84] and overexpression of amyloid precursor protein (APP) and its metabolite Aβ, which are detected as early as 21 gestational wk [49, 87]. Intraneuronal Aβ has been reported as early as 1 year [81] with accumulations of Aβ peptides appearing between the ages of 8 to 12 years [12, 30]. Although phospho-tau has been found intra-neuronally at early ages in individuals with DS, fully mature NFTs do not appear until the fourth decade of life [12, 31]. Fetal APP, with its gene located on chromosome 21, has been implicated in neurogenesis, neuronal differentiation and synaptogenesis during neurotypical development [23, 64], is increased and associated with alterations in GABAergic systems in development [58] and adulthood in DS [15]. Additionally, human phosphorylated fetal tau (N03R), found in distal portions of growing axons, is downregulated after axons reach their target sites before birth [80] and perturbed early in the maturation of the DS brain [57]. Although evidence suggests that changes in prenatal brain development disrupt the function and structure of cortical areas in DS, there is a lack of information regarding postnatal cortical abnormalities in DS. Defining the alterations in cortical neuronal differentiation and cyto- and chemical architecture [75, 82] is crucial for a better understanding of cortical neuronal circuitry and neurotransmission during the postnatal period of brain development in DS.

The effects of trisomy 21 on postnatal FC maturation remain under-investigated. Here, we examined postnatal differentiation of neuronal profiles using quantitative immunohistochemistry for the intermediate cytoskeletal non-phosphorylated high-molecular-weight neurofilament (NHF) proteins; the GABAergic interneuron calcium-binding proteins (CBP) Calbindin D-28K (Calb), Calretinin (Calr) and Parvalbumin (Parv); the neuronal microtubule-associated protein doublecortin (DCX); and the cellular proliferation marker Ki-67 applied to FC tissue obtained postmortem from 28-wk gestation to 3-year-old DS and NTD cases. In addition, we also examined the presence of Aβ and phosphorylated tau proteins using antibodies to APP/Aβ (6E10), Aβ_1–42_, PHF-1, and CP13.

## Subjects, materials and methods

### Subjects and tissue samples

Postmortem FC tissue was obtained from 11 male and 8 female cases ranging from premature (31 gestational wk) to 196 wk with DS (n = 10) and age-matched neurotypical (NTD) controls (n = 9). DS tissue was acquired from Phoenix Children’s Hospital (PCH) (n = 4) and Ann & Robert H. Lurie Children's Hospital of Chicago (LCH) (n = 6), while NTD samples were obtained from PCH (n = 9). Tissue was processed according to IRB guidelines meeting exemption criteria in 45 CFR 46.101 (b) and managed under Barrow Neurological Institute recommendations.

Sex, age at birth and death, postnatal life between birth and death, brain weight, body weight, height (measured crown to heel), postmortem interval (PMI) and cause of death/comorbidity are reported in Table 1. Additionally, tissue was examined from 1 NTD and 3 DS premature infants, who died prior to 40 wk of gestation, which is considered a full-term pregnancy/infant [83] (Table 1). To take into account the developmental stage of premature (preterm) infants, age consisted of combining the number of gestational wk at birth plus the number of postnatal wk of life. In all DS cases, trisomy 21 was confirmed at each institution using standard peripheral blood lymphocyte karyotyping procedures. Independent of tissue source, all brains were fixed in 10% neutral buffered formalin and embedded in paraffin. Blocks containing the FC were sectioned at 4 µm (DS-PCH) and 8 µm (DS-LCH/Control-PCH) thickness on a Minot microtome, mounted on charged slides and stored at room temperature until processing.

**Table 1:** Demographics for NTD and DS subjects

ID	Sex	Age at birth (wk)	Age at death (wk)	Postnatal life (wk)	Brain weight (g)	Body weight (kg)	Height (cm)	PMI (hr)	Tissue Source	Cause of death/comorbidity
*NTD*										
C1	M	27.9	28.0*	0.1	160.4	2.4	38.2	46	PCH	Non-immune hydrops
C2	F	38.0	38.1	0.1	360.0	2.6	48.5	–	PCH	Pulmonary hemorrhage
C3	F	37.0	41.0	4.0	373.3	3.6	55.5	6	PCH	Congenital heart disease
C4	F	39.0	42.0	3.0	420.9	5.5	39.0	16	PCH	Congenital heart disease
C5	M	40.0	44.7	4.7	518.0	3.7	54.5	26	PCH	Congenital heart disease
C6	M	39.0	48.0	9.0	584.2	5.7	55.8	26	PCH	Congenital heart disease
C7	M	40.0	51.0	11.0	670.0	6.4	63.5	17	PCH	Septicemia
C8	M	40.0	83.5	23.5	1134.9	9.5	77.8	29	PCH	Acute pneumonia
C9	M	40.0	174.7	135.0	1103.0	13.1	90.1	18	PCH	Lymphoma
*DS*										
DS1	F	30.7	31.6*	0.9	210.0	3.0	44.5	76	PCH	Myeloproliferative disorder
DS2	M	31.0	32.9*	1.9	176.0	3.3	42.5	42	LCH	Bronchopulmonary dysplasia
DS3	F	31.7	33.1*	1.4	209.0	2.1	42.0	12	LCH	Transient abnormal myelopoiesis
DS4	M	31.7	40.7	9.0	381.0	2.5	49.0	58	LCH	Congenital cardiac anomalies
DS5	F	40.0	44.0	4.0	243.0	3.0	49.3	20	LCH	Congenital cardiac anomalies
DS6	F	37.7	45.7	8.0	445.0	5.2	55.0	16	LCH	Congenital cardiac anomalies
DS7	M	40.0	53.0	13.0	330.0	4.1	49.0	21	LCH	Interstitial lung disease
DS8	M	40.0	57.4	17.4	630.0	5.6	48.0	14	PCH	Congenital heart disease
DS9	F	35.0	191.4	156.4	834.5	13.5	90.3	39	PCH	Congenital heart defects
DS10	M	40.0	196.4	156.4	1163.0	14.4	97.0	22	LCH	Diffuse alveolar damage

*Premature infants, PMI: postmortem interval, PCH: Phoenix Children’s hospital, LCH: Lurie Children’s Hospital

### Immunohistochemistry

Two cortical sections from each case were deparaffinized, rehydrated in a descending series of ethanol concentrations (100%, 95%, 70%, and 50%) and pretreated either with a citric acid (pH 6) solution for 10 minutes (min) in a microwave or with 88% formic acid for 10 min to detect APP/Aβ and Aβ_1–42_. Immunohistochemistry was performed as previously described [60] using antibodies directed against non-phosphorylated heavy-molecular-weight neurofilament peptide (SMI-32), which is highly expressed in human neocortical layers III and V pyramidal neurons [11, 18, 36, 55], DCX (a marker of neurogenesis), and Ki-67 (a nuclear marker of proliferation/cell division) [20, 77]. APP/Aβ (6E10), Aβ_1–42_, CP13, PHF-1, and CBPs (Calb, Calr and Parv). Sections were washed and incubated with a primary antibody (see Table 2) in a Tris-buffered saline (TBS) Triton X-100/1% goat serum (GS) solution overnight at room temperature. After three 1% GS TBS washes, the sections were incubated with a goat anti-mouse or anti-rabbit biotinylated secondary antibody (1:200) for 1 h (hr) (Vector Labs, Burlingame, CA) based upon the appropriate primary antibody, followed by Vectastain ABC kit (1 h) (Vector Labs) incubation. Subsequently, sections were developed in acetate-imidazole buffer solution containing 0.05% 3,3′-diaminobenzidine tetrahydrochloride (DAB) (Thermofisher Scientific, Waltham, MA) and 0.005% hydrogen peroxide. To enhance immunostaining for CP13, sections were developed using a solution consisting of DAB and nickel sulfate (0.5%) resulting in a blue-black precipitate. Slides were then dehydrated in an ascending series of ethanol concentrations (50%, 70%, 95%, and 100%), cleared in xylenes, and coverslipped using DPX (Electron Microscopy Sciences, Hatfield, PA). An immunostained section per case was counterstained using Mayer’s Hematoxylin for 1.5 min, washed under running tap water for 4 min, soaked in a bluing solution for 20 s, washed in distillated water, dehydrated, cleared in xylenes, and coverslipped using DPX. To control for batch-to-batch variation, sections from each case were processed simultaneously for each antibody. Controls consisted of the omission of primary antibodies resulting in a lack of immunoreactivity. To test the specificity of 6E10 immunostaining, this antibody was preabsorbed against purified human Aβ_1–17_ (AnaSpec Inc., Fremont, CA) at a concentration of 50–500 µg/μl overnight, followed by immunolabeling of postnatal cortical tissue according to the above protocol, resulting in a reduction of immunoreactivity in both groups. Additionally, FC paraffin embedded tissue from an 82-year-old female with neuropathologically confirmed AD (Braak NFT stage VI) and an anaplastic astrocytoma tumor (8-year-old female) were used as positive controls for tau [80], Aβ and Ki-67 antibody staining.

**Table 2:** Antibody characteristics

Antigen	Primary antibody	Dilution	Company Cat. #	Secondary Antibody (Company)
SMI-32*	Mouse monoclonal to anti-Neurofilament H, non-phosphorylated	1:500 1:50*	Biolegend 801701	Biotinylated goat anti-mouse IgG (Vector Laboratories) Cy3 donkey anti-mouse IgG (Jackson Immunoresearch Laboratories)*
APP/Aβ	Mouse monoclonal to residues 1–16 of Aβ (6E10)	1:300	Biolegend 803002	
Parvalbumin	Mouse monoclonal anti-parvalbumin	1:500	Millipore MAB1572	
Doublecortin	Mouse monoclonal neuronal migration protein DCX E-6	1:250	Santa Cruz Biotechnology sc-271390	
CP13	Mouse monoclonal phospho-tau (Ser202)	1:100	Gift from Peter Davies	
PHF-1	Mouse monoclonal phospho-tau (Ser396/Ser404)	1:100	Gift from Peter Davies	
Ki-67	Mouse monoclonal to human MIB-1	1:500	Dako M7240	
Calbindin D28-K*	Rabbit polyclonal to 28 kD calcium-binding protein	1:1000 1:75*	Swant CB38	Biotinylated goat anti-rabbit IgG (Vector Laboratories) Cy5 donkey anti-rabbit (Jackson Immunoresearch Laboratories)*
Calretinin	Rabbit polyclonal to 99 aa epitope from the internal region of rat calretinin	1:500	Millipore AB5054	
Aβ_1–42_	Rabbit polyclonal to 6 aa peptide sequence from C-terminus of human Aβ_1–42_	1:100	Millipore AB5078P	

*Immunofluorescence staining

### Immunofluorescence

Sections were deparaffinized, hydrated, and pretreated with heated citric acid (pH 6) in a microwave for 10 min. Sections were then incubated simultaneously with a mouse monoclonal against SMI-32 (1:50, Biolegend, San Diego, CA) and a rabbit polyclonal against Calb (1:75, Swant, Marly, Switzerland) in a solution containing TBS 0.25% Triton X-100/1% donkey serum overnight at room temperature. After three washes in TBS/1% donkey serum solution, sections were incubated with a donkey anti-mouse Cy3 secondary antibody (1:300, Jackson Immunoresearch Laboratories, Inc., Chester County, PA) for 1 h followed by TBS washes and incubated with a donkey anti-rabbit Cy5 secondary antibody (1:200, Jackson Immunoresearch Laboratories, Inc., Chester County, PA) for 1 h. After several washes in TBS, sections were incubated with the nuclear marker DAPI (D1306, Invitrogen, Carlsbad, CA) at 1:2000 concentration for 10 min. Subsequently, slides were washed and coverslipped using Invitrogen Prolong Glass Antifade Mountant (Invitrogen, Carlsbad, CA). Immunofluorescence images were captured using an Echo Revolve Fluorescence microscope (San Diego, CA).

### Histochemistry

To examine FC cytoarchitecture, two additional slides from each case were stained using thionin, a Nissl stain that visualizes the neuronal endoplasmic rough reticulum and nucleus [40]. Sections were deparaffinized, placed in 100% ethanol for 3 min then a 50% chloroform/50% ethanol solution for 15 min, rehydrated in a decreasing gradient of alcohols, soaked in a 0.5% thionin solution (pH 4.3) for 7 min, washed with distilled water, dehydrated in an increasing gradient of alcohols, cleared in xylenes, and coverslipped using DPX (Electron Microscopy Sciences, Hatfield PA) [60]. To control for batch-to-batch variation, sections from each case were processed at the same time, masked to demographics.

### Cell quantitation

Tissues stained for thionin, SMI-32, DCX, Calb, Calr, Parv, APP/Aβ (6E10), Aβ_1–42_, CP13, and PHF-1 were imaged using a Nikon Eclipse 80i and analyzed using NIS-Elements BR software. All cell density counts were performed in 10 different areas on each slide at 400 × magnification within SG layers II-III and IG layers V-VI, except for Calb which was counted at 200 × magnification (due to the low density of positive cells) and an average cell count was calculated for each layer per case. Additionally, all counts were normalized against thionin-stained cell numbers. APP/Aβ plaque number and load were examined at 100 × magnification and plaque load was calculated as a percentage of the area immunostained versus the area examined of 1.02 mm^2^ using NIS-Elements BR software. Counts were performed by an investigator blind to case demographics.

### Statistical analysis

Non-parametric statistics were used for all analyses due to small sample sizes for the AD and NTD groups (n ≤ 10) and that assumptions of normality and equality of variance were not met for any of the numeric dependent variables. Neuronal counts and case demographics were compared between groups using a non-parametric Mann–Whitney rank sum test, a Wilcoxon signed rank test and Fisher exact test (SigmaPlot 14.0, Systat Software, San Jose, CA). Statistical significance level (*p*) was set at less than 0.05 (two-tailed). Correlations for within-group cell count variables were performed using a Spearman’s rank correlation and false discovery rate (FDR) was applied to control for Type I error when conducting multiple comparisons. Cell counts were graphically presented using boxplots, histograms and dot plots and correlations were represented as linear regressions (SigmaPlot 14.0, Systat Software, San Jose, CA).

## Results

### Case demographics

There were no significant differences in age, brain weight, height (measured from crown to heel), or body weight between DS and NTD groups (Mann–Whitney rank sum test; *p* > 0.05, Table 3). Average age was 61.22 wk (range, 28–174) for NTD and 72.63 wk (range, 31–196) for DS; average brain weight was 591.63 g (range, 160–1134) for NTD and 462.15 g (range, 176–1163) for DS; average height was 58.10 cm (range, 38–90) for NTD and 56.66 cm (range, 42–97) for DS; average body weight was 5.82 kg (range, 2–13) for NTD and 5.66 kg (range, 2–14) for DS. No significant differences were found for PMI (Mann–Whitney rank sum test; *p* > 0.05) or sex (Fisher exact test; *p* > 0.05) between groups.

**Table 3:** Summary of case demographics

	NTD n = 9	DS n = 10	*p* value
Age (wk)	61.22 ± 15.06* Min. 28.00; Max. 174.70	72.63 ± 20.40 Min. 31.57; Max. 196.43	ns^a^
Brain weight (g)	591.63 ± 110.85 Min. 160.40; Max. 1134.90	462.15 ± 102.19 Min. 176.00; Max. 1163.00	ns^a^
Height (cm)	58.10 ± 5.67 Min. 38.20; Max. 90.10	56.66 ± 6.29 Min. 42.00; Max. 97.00	ns^a^
Body weight (kg)	5.82 ± 1.17 Min. 2.40; Max. 13.10	5.66 ± 1.43 Min. 2.10; Max. 14.40	ns^a^
PMI (hr)	23.00 ± 4.19 Min. 6.00; Max. 46.00	32.00 ± 6.75 Min. 12.00; Max. 76.00	ns^a^
Sex M (%)/F (%)	5 (66.66%)/4 (33.33%)	5 (50.00%)/5 (50.00%)	ns^b^

^a^Mann–Whitney rank sum test

^b^Fisher exact test, ns; not significant

*Mean ± standard error (SE)

### Postnatal FC cytoarchitecture

**Fig. 1:**
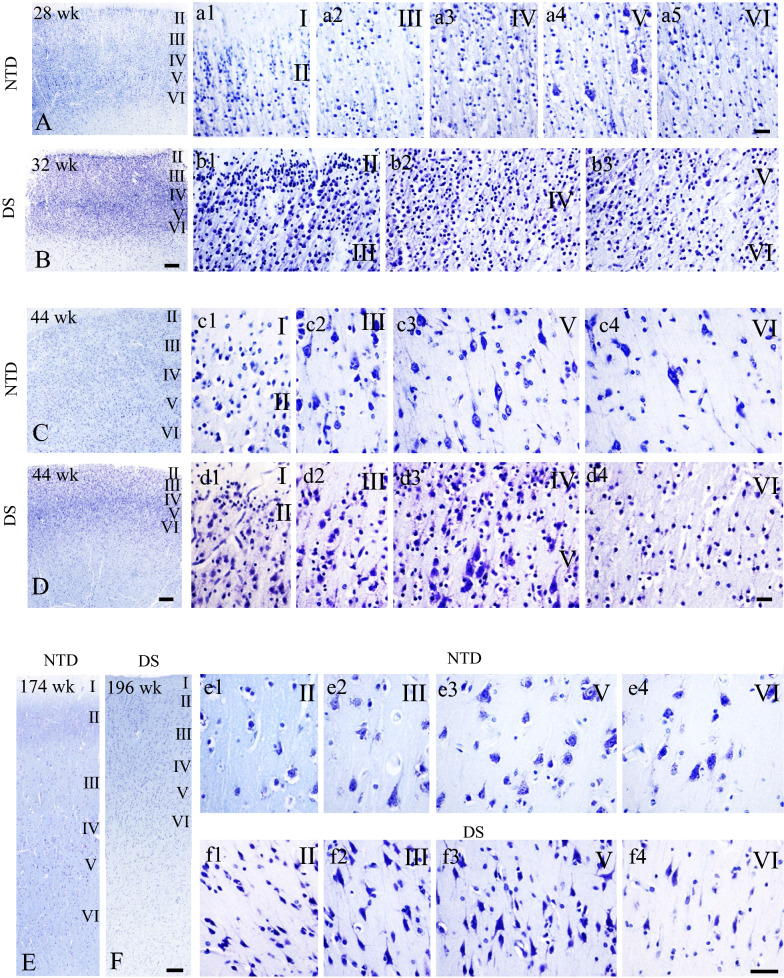
Low-magnification images of thionin-stained FC showing lamination patterns in 28 (**A**), 44 (**C**), and 174 (**E**) wk in NTD and 32 (**B**), 44 (**D**), and 196 (**F**) wk in DS. Layers I-VI were also imaged at a higher magnification for each NTD (28 wk: **a1**–**a5**; 44 wk: **c1**–**c5**; 174 wk: **e1**–**e5**) and DS (32 wk: **b1**–**b5**; 44 wk: **d1**–**d5**; 196 wk: **f1**–**f5**) case showing cell type and cellular distribution. Note the presence of pyramidal neurons in layer V at 28 wk in a premature NTD infant, while lacking in a 32 wk DS infant, as well the higher cell density in layers II to VI in DS than in NTD at all ages. Scale bar in **B**, **D** and **F** = 200 µm and applies to **A**, **C** and **E**; **a5**, **d4** and **f4** = 25 µm applies to **a1**–**b2**, **c1**–**d3** and **e1**–**f3**, respectively

Thionin stained sections were used to examine the lamination and cytoarchitecture of the FC from DS cases aged 31 to 196 wk compared to 28 to 174 wk NTD infants and children (Fig. 1). Although FC thionin-stained sections revealed the appearance of a six-layered cortex, cortical lamination was better differentiated at NTD ages 28 to 174 wk, than in DS cases (Fig. 1A–F). Despite the low intensity of thionin cytoplasmic staining seen in the youngest NTD case (28 wk), the FC displayed a developing isocortical lamination pattern consisting of non-differentiated cellular profiles displaying apical processes. In NTD, layer V displayed large pyramidal-shaped neurons (Fig. 1a1, a4). By contrast, at 32 wk the DS cortex displayed more intense thionin neuronal staining with more compact cortical layers (Fig. 1B). Layer II displayed dense clusters of strongly stained cells (Fig. 1B, b1) but layer V lacked the appearance of large pyramidal neurons as seen in the 28 wk NTD case (Fig. 1b3). Interestingly, at 32 and 44 wk we found a distinct layer IV in DS (Fig. 1B, D), which was not observed in NTD (Fig. 1C). At 44 wk, cellular differentiation, particularly in pyramidal cells in layers III and V, was evident in both NTD and DS cases (Fig. 1c2, c3, d2, d3). Layer VI showed fusiform neurons at 44 wk in NTD (Fig. 1c4), but not in DS. At this age, as well as at 196 wk, the lamination of the DS cortex (Fig. 1D, F) was still less well-differentiated compared to NTD (Fig. 1C, E). In the oldest (196 wk) DS case, a less organized cortex containing a higher density of small undifferentiated cellular profiles was observed compared to the oldest 174 wk NTD case. At this age, layer III and V neurons had a more typical pyramidal shape in NTD (Fig. 1E, F).

**Fig. 2:**
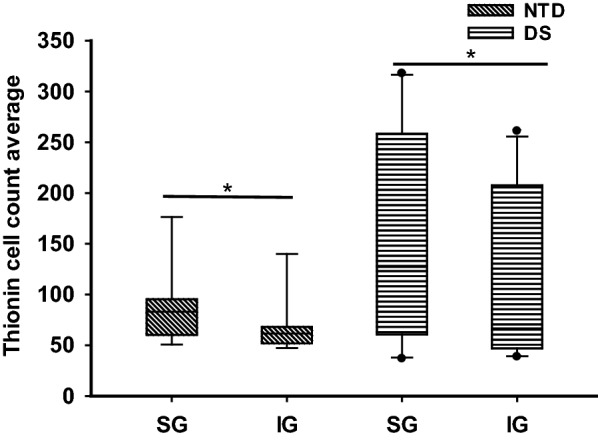
Boxplot showing thionin-positive cell count average in the SG (II–III) and IG (V–VI) lamina in NTD (n = 9) and DS (n = 10). Statistical analysis revealed a significantly greater number of cells in SG compared to IG for both NTD and DS (Wilcoxon signed rank test, NTD *p* = 0.004; DS *p* = 0.027). No significant differences were found in the SG and IG layers between groups (Mann–Whitney rank sum test). *p < 0.05

**Fig. 3:**
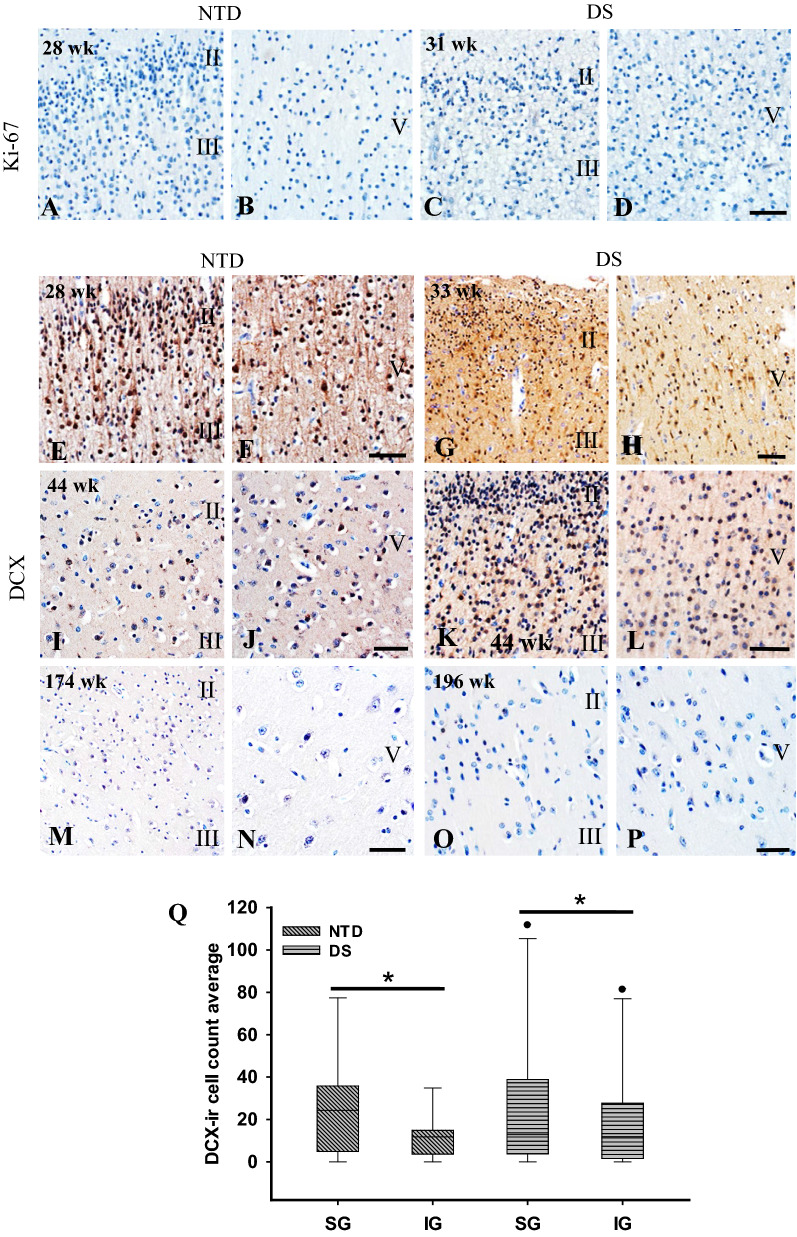
Images showing the absence of Ki-67 immunostaining in the SG and IG layers in a 28 wk NTD (**A**, **B)** and a 31 wk DS (**C**, **D**). Photos of DCX- immunoreactivity in cortical SG and IG layers in NTD aged 28 **(E**, **F**), 44 (**I**, **J)** and 174 wk (**M**, **N**) and DS aged 33 (**G**, **H**), 44 **(K**, **L**), and 196 (**O**, **P**) wk subjects. Note that DCX immunostaining decreased with age and was absent in the oldest cases in both groups (**M**–**P**). Small undifferentiated cells were DCX positive in 28–44 wk NTD (**E**, **F**, **I**, **J**) and 33–44 wk DS (**G**, **H**, **K**, **L**) cases. Note many more DCX positive cells in SG and IG layers at DS 44 wk compared to a NTD 44 wk suggesting a delay in neuronal migration/neurogenesis in DS. Boxplot showing a significantly greater number of DCX positive cells in SG compared to IG in both NTD (n = 9) and DS (n = 10) (**Q**) (Wilcoxon signed rank test, NTD *p* = 0.008; DS *p* = 0.008), while no significant differences were found in the SG and IG layers between groups (Mann–Whitney rank sum test). *p < 0.05. Scale bars: D = 50 µm applies to **A**–**C**; F = 50 µm applies to **E**; H = 50 µm applies to **G**; J = 50 µm applies to **I**; L = 50 µm applies to **K**; N = 50 µm applies to **M**; and P = 50 µm applies to **O**

Quantitation of thionin-stained cells in SG (II-III) and IG (V-VI) layers revealed no significant differences between groups. A within group analysis found significantly greater cell numbers in SG compared to IG in both NTD (Wilcoxon signed rank test, *p* = 0.004) and DS (Wilcoxon signed rank test, *p* = 0.027) (Fig. 2).

### Postnatal FC proliferation and neurogenesis

Sections immunostained for Ki-67, a nuclear protein expressed during cell division that marks cell proliferation, did not reveal positive profiles at any age in either group (e.g., Fig. 2A–D). DCX, a microtubule-associated protein highly expressed in neuroblasts/immature neurons, was used to reveal neurogenesis and neuronal migration in the postnatal FC. DCX-ir cells were observed in SG and IG layers from 28 to 83 wk in NTD (Fig. 3E, F, I, J) and from 31 to 53 wk in DS (Fig. 3G, H, K, L). In the youngest cases from both groups (28 to 44 wk NTD, 33 to 44 wk DS), DCX immunoreactivity was most prominent in small undifferentiated cells within SG layers II-III (Fig. 3E–L), which displayed immunoreactivity in leading apical processes. Notably, layers II-III and V-VI showed stronger DCX immunoreactivity in DS compared to NTD at 44 wk (Fig. 3I–L), but no immunostaining was seen in the oldest cases in either group (Fig. 3M–P). These findings suggests that neurogenesis is completed earlier and plays a role in FC dysfunction in DS.

Quantitation revealed no significant difference in DCX-ir cell numbers in SG and IG layers between NTD and DS (Mann–Whitney rank sum test, *p* > 0.05). By contrast, a within group analysis revealed a significantly greater number of DCX-ir cells in SG compared to IG layers (Wilcoxon signed rank test, *p* = 0.008) (Fig. 3Q). When data were normalized to thionin counts, similar findings were found in NTD, but not DS cases. Moreover, we did not find a significant difference in DCX-ir cell number between SG and IG layers.

### Postnatal FC NHF profiles

**Fig. 4:**
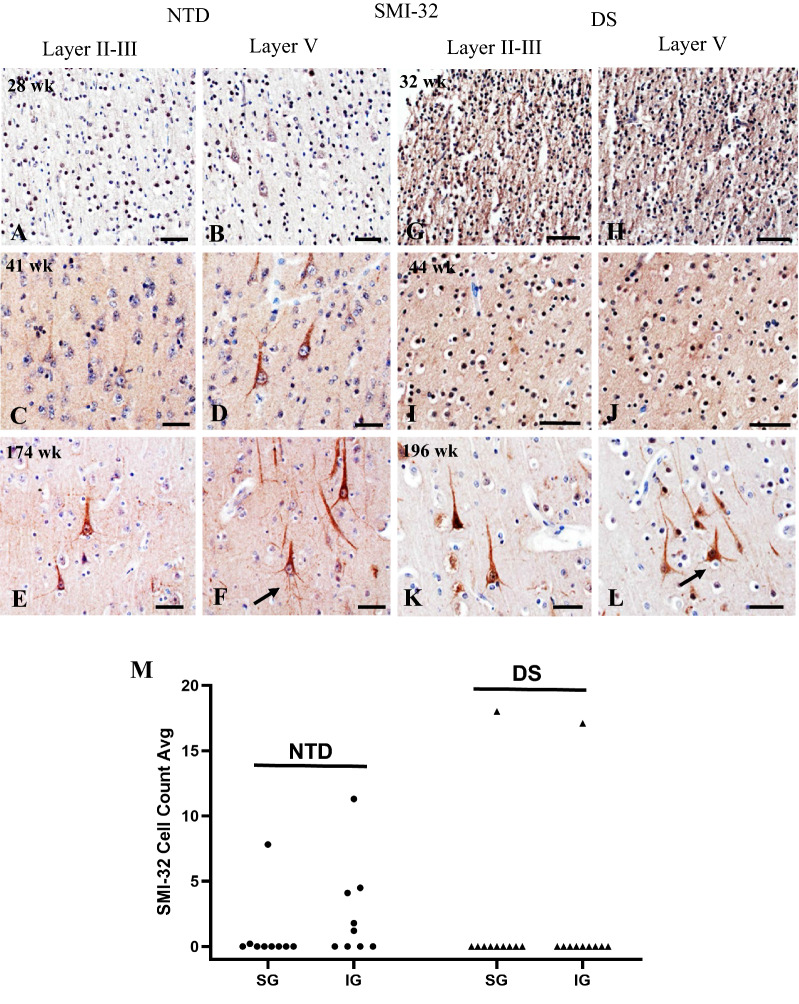
Images showing SMI-32 immunostaining in NTD at 28 (**A**, **B**), 41 (**C**, **D**), and 174 wk (**E**, **F**) and in DS at 32 (**G**, **H**), 44 (**I**, **J**), and 196 wk (**K**, **L**). In the youngest 28 wk NTD case, lightly labeled SMI-32-ir cells pyramidal were found in layer V (**A**, **B**). Note the increase in SMI-32 immunoreactivity at 32 weeks in DS (**G**, **H**). At 41 and 44 wk, SMI-32-ir pyramidal cells were observed in layers III and V in NTD (**C**, **D**), whereas none were seen in DS (**I**, **J**). In the oldest cases, 174 wk NTD and 196 wk DS, strong SMI-32-ir pyramidal cells with apical processes were observed in layers III and V (**E**, **F**, **K**, **L**). Note the presence of many more basal process (arrows) in SMI-32 positive pyramidal neurons in layer V in the oldest 174 wk NTD compared to 196 wk DS case. **M** Dot plot showing no significant differences in SMI-32-ir cell counts between groups or within groups between SG and IG layers (NTD: n = 9, DS: n = 10) (Mann–Whitney rank sum test, Wilcoxon signed rank test). Notably, the IG area (V–VI) in NTD showed the highest SMI-32-ir cell count. Scale bars = 50 µm

SMI-32 detects an intermediate non-phosphorylated neurofilament protein highly expressed in the soma and dendrites of mature neocortical pyramidal neurons [11, 18, 36, 55], which marks neuronal maturation (Fig. 4). Although we observed greater neuropil SMI-32 immunoreactivity at 32 wk in DS compared to 28 wk in NTD (Fig. 4G, H), only a few SMI-32-ir pyramidal-shaped cells were found in layer V in NTD (Fig. 4B). From 41 wk and onwards, pyramidal neurons were observed in layers III and V showing strong immunoreactivity in apical and basal processes, exclusively in NTD (Fig. 4C–F). By contrast, only the oldest DS case (196 wk) contained SMI-32-ir pyramidal cells in layers III and V (Fig. 4K, L).

Although a greater number of SMI-32-ir cells were present within layer V in NTD compared to DS, no significant differences in neuronal counts were observed between groups when comparing SG and IG layers (Fig. 4M) (Mann–Whitney rank sum test, *p* > 0.05). In addition, no significant differences were found between the SG and IG counts within groups (Fig. 4M) (Wilcoxon signed rank test, *p* > 0.05), similar to that found when data were normalized to thionin cell counts.

### Postnatal CBP reactivity in the FC

**Fig. 5:**
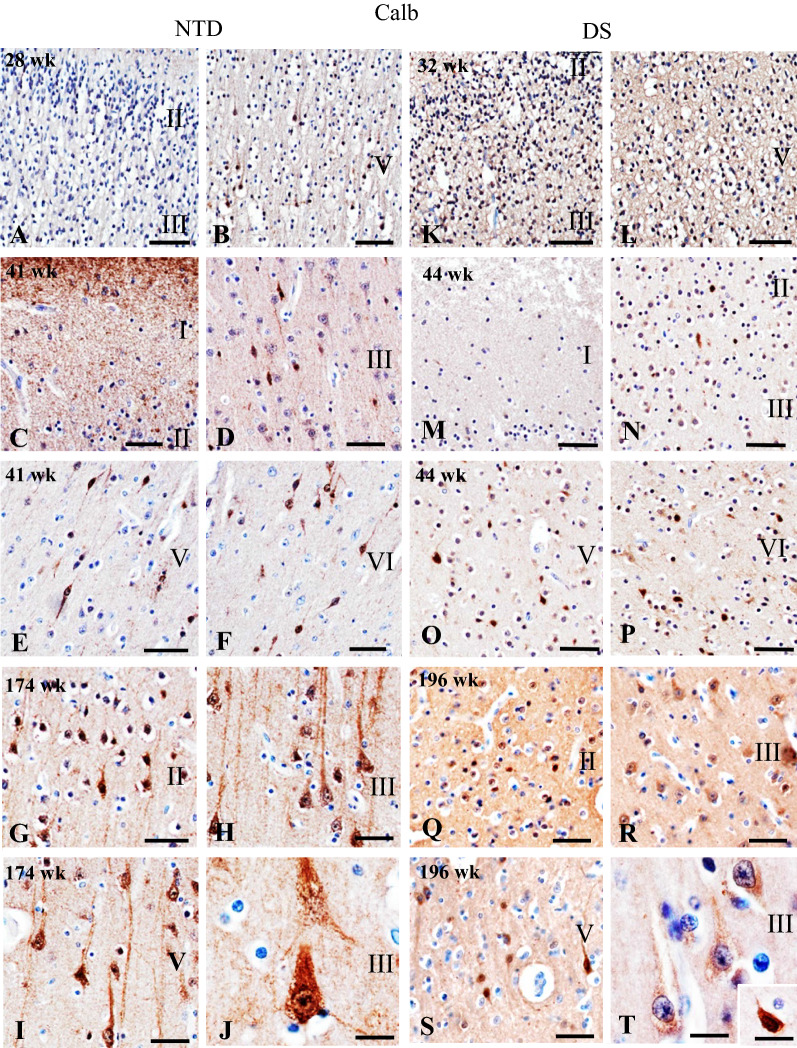
Images showing Calb immunostaining in NTD at 28 (**A**, **B**), 41 (**C**–**F**), and 174 (**G**–**J**) wk and in DS at 32 (**K**, **L**), 44 (**M**–**P**) and 196 (**Q**–**T**) wk. A small number of Calb-ir cells with pyramidal shape were visible in layer V in a 28 wk NTD (**B**) compared to extensive Calb-ir neuropil. Calb positive pyramidal cells in SG and IG layers at 32 wk in DS (**K**, **L**). At 41 weeks in NTD, Calb immunoreactivity was seen in layer I (**C**), in small cells in layers II/III (**C**, **D**) and fusiform neurons in layers V/VI (**E**, **F**). At 44 weeks in DS, Calb immunostaining was only observed in small cells in layers II/III (**N**) and V/VI (**O**, **P**). In the oldest cases (174 wk NTD and 196 wk DS), Calb-ir interneurons and pyramidal cells were seen in layer II (**G**, **Q**), while strong cytoplasmic Calb immunoreactivity was seen in pyramidal cells in layer III (**H**, **J**, **R**, **T**) and layer V (**I**, **S**). Inset in **T** shows a higher magnification image of Calb-ir interneuron in layer III at 196 weeks in DS. Note greater Calb immunoreactivity in the pyramidal apical process in the oldest NTD (**H**–**J**) compared to the oldest DS case (**R**–**T**). Scale bars: A–I = 50 µm; J, T and inset = 20 µm; K–S = 50 µm

Calb, Calr, and Parv were used to visualize the development of non-pyramidal interneurons in the FC. Microscopic analysis revealed a few undifferentiated Calb-ir cells in layer V in the youngest NTD infant (28 wk) compared to extensive immunoreactivity throughout the cortical neuropil, but not in cells, in the youngest DS infant (33 wk) (Fig. 5A, B, K, L). At 41 wk in NTD, we found a band of Calb-ir in the external portion of layer I, positive cells in layers II/III and fusiform-appearing neurons in layers V/VI (Fig. 5C–F). Although layer I was not Calb positive, small oval-shaped Calb-ir cells were scattered in layers II/III and V/VI at 44 wk in DS (Fig. 5M–P). In both the oldest NTD (174 wk) and DS (196 wk) cases, layer II showed Calb-ir interneuronal and pyramidal cells, while layers III and V displayed strongly immunostained pyramidal-shaped perikarya and apical dendrites (Fig. 5G–J, Q–T). Calb-ir apical processes were more evident in the oldest NTD compared to the oldest DS case (Fig. 5J, T).

**Fig. 6:**
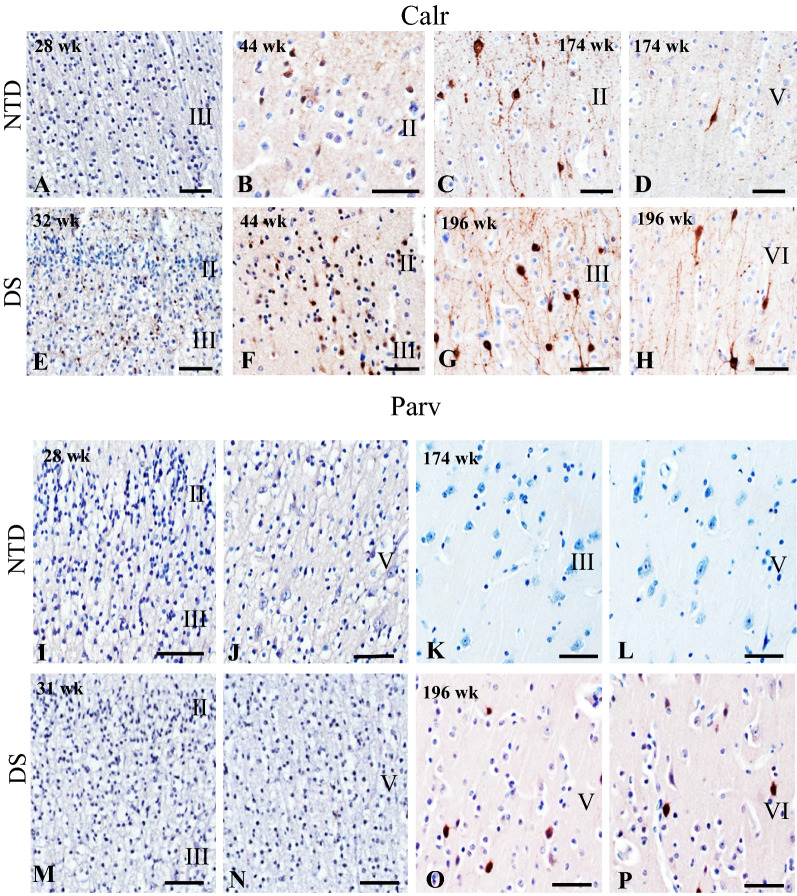
Images showing Calr immunostaining in NTD aged 28 (**A**), 44 (**B**), and 174 (**C**, **D**) wk and in DS at 32 (**E**), 44 (**F**), and 196 (**G**, **H**) wk. No positive cells were observed in 28 wk NTD (**A**), while small neurons displayed staining at 44 wk NTD in layers II/III (**B**). In 174 wk NTD, large bipolar fusiform cells were observed in layers II/III (**C**) and layer V (**D**). A 32 wk DS case displayed small Calr-ir cells in layers II/III (**E**). Note that the cellular processes stained more intensely in layers II/III at 44 wk (**F**). In a 196 wk DS case, fusiform Calr-ir cells were observed in layer III (**G**) and layers V/VI (**H**). Images showing the absence of Parv immunoreactivity in FC SG and IG layers in 28 and 174 wk NTD (**I**–**L**) and the youngest 31 wk DS cases (**M**–**N**). Small and intensely stained Parv-ir cells were seen in layers V/VI in the oldest 196 wk DS case (**O**, **P**). Scale bars = 50 µm

In NTD, Calr-ir cells were not seen in the FC until 44 wk of age (Fig. 6A, B). At this age, numerous small cells were observed in layers II/III (Fig. 6B). In the oldest NTD case (174 wk), larger bipolar fusiform-shaped Calr-ir perikarya were observed in layers II/III and V (Fig. 6C, D). In contrast, as early as 32 wk, Calr-ir cells were observed in layers II/III in DS (Fig. 6E). At 44 wk in DS, layer II/III Calr-ir neurons appeared larger in size and more abundant (Fig. 6F). At wk 196 in DS, fusiform-shaped Calr-ir cells were found in layers II/III and V/VI (Fig. 6G, H).

Unlike Calb and Calr, Parv immunoreactivity was not detected in the youngest DS and NTD cases (Fig. 6I, J, M, N). The first Parv-ir cells were seen in layers V/VI in the oldest DS case (196 wk) (Fig. 6O, P), but not in NTD (174 wk) (Fig. 6K, L).

**Fig. 7:**
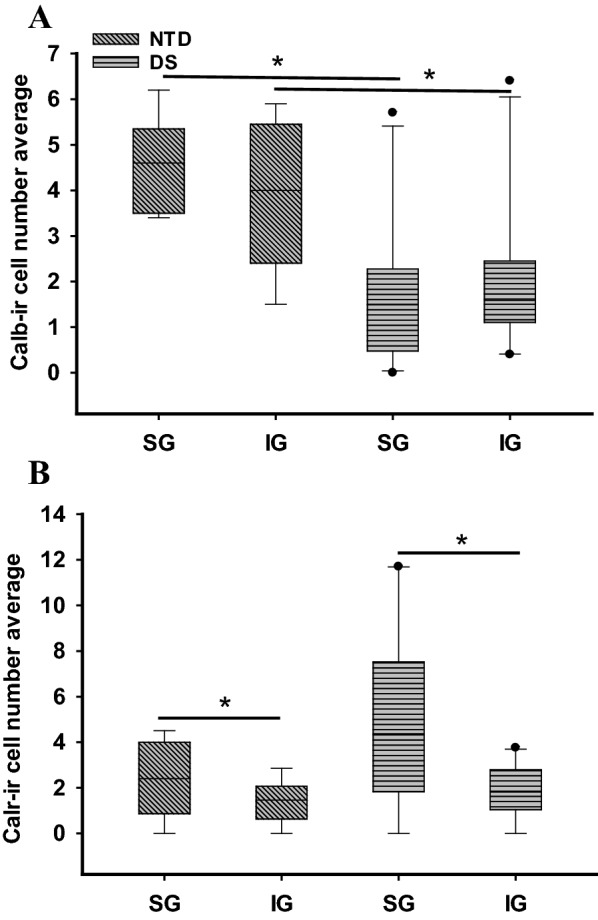
Boxplots showing significantly higher Calb-ir cell number in NTD (n = 9) compared to DS (n = 10) in both the SG and IG regions (Mann–Whitney rank sum test, SG *p* = 0.003; IG *p* = 0.024) (**A**), while Calr-ir counts were significantly higher in SG compared to IG in both NTD and DS (Wilcoxon signed rank test, NTD *p* = 0.016; DS *p* = 0.008) (**B**). *p < 0.05

Quantitation revealed that Calb-ir cell number in SG (Mann–Whitney rank sum test, *p* = 0.003) and IG (Mann–Whitney rank sum test, *p* = 0.024) layers were significantly higher in NTD compared to DS (Fig. 7A). In contrast, there were no significant differences in Calb-ir cell number between SG and IG layers within groups (Wilcoxon rank signed test, *p* > 0.05) (Fig. 7A) and normalized data revealed no significant changes in Calb-ir cell numbers in SG and IG within groups or between groups. Unlike Calb, there were no significant differences in Calr-ir cell numbers between NTD and DS in any layer (Mann–Whitney rank sum test, *p* > 0.05) (Fig. 7B). Calr-ir counts revealed a significantly greater cell density in SG compared to IG in NTD (Wilcoxon signed rank rest, *p* = 0.016) and in DS (Wilcoxon signed rank test, *p* = 0.008) (Fig. 7B). Although normalization of the data yielded similar findings for NTD, there were no significant differences in Calr-ir cell density between the SG and IG layers in DS. Due to the limited number of Parv positive profiles, statistical analysis was not performed.

### FC APP/Aβ, Aβ_1–42_ and phosphorylated tau immunoreactivity

**Fig. 8:**
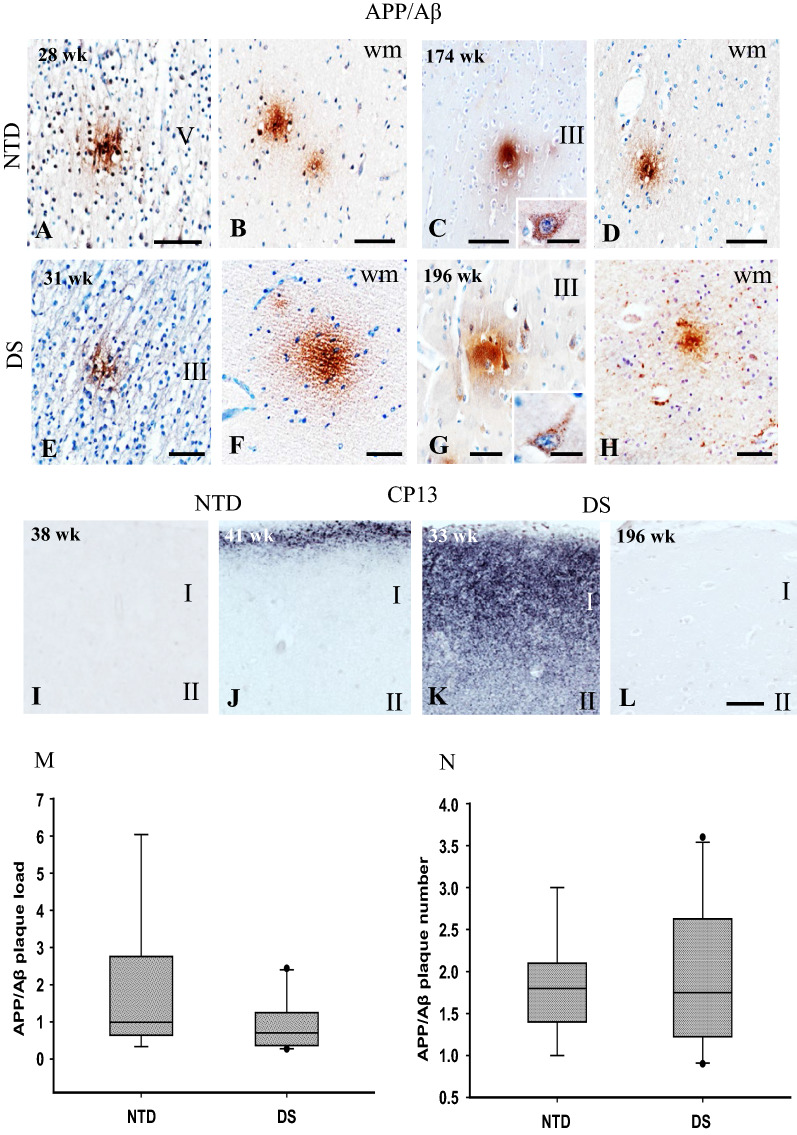
Images of the FC showing APP/Aβ-ir deposits in gray and white matter in NTD at 28 and 174 wk (**A**–**D**) and in DS 31 and 196 wk cases (**E**–**H**). Insets **C** and **G** show APP/Aβ-ir pyramidal neurons in 174 wk NTD (**C**) and 196 wk DS (**G**). **I**–**L**. Tissue in panels A to H were counterstained with hematoxylin. Images showing CP13 immunostaining in NTD at 38 and 41 wk and in DS at 33 and 196 wk. Granular CP13 immunoreactivity was observed in layer I in 41 wk NTD (**J**) and 33 wk DS (**K**) cases, but no CP13-ir cells were detected (**I**–**L**). **M**–**N**. Boxplots showing no significant differences in plaque load (**M**) and plaque counts (**N**) between NTD (n = 9) and DS (n = 10) groups (Mann–Whitney rank sum test). Abbreviation: wm, white matter. Scale bars: A–L = 50 µm, **L** applies to **I**–**K**, insets in **C** and **G** = 10 µm

To reveal the presence of Aβ plaques in the FC, we used antibodies that detect either APP/Aβ (6E10) or Aβ_1–42_, the main component of neuritic/senile plaques resulting from the concerted cleavage of APP by β- and γ-secretase. We found 6E10-ir accumulations, similar to diffuse plaques, in the FC gray and white matter at all ages with no specific laminar distribution in either group (Fig. 8A–H). APP/Aβ-ir granules were observed in the cytoplasm of pyramidal-shaped cells only in the oldest NTD (174 wk) and DS (196 wk) cases (Fig. 8C, G). Conversely, Aβ_1–42_ immunoreactivity was not observed in the FC in either group, suggesting that non-amylogenic APP derivatives comprise these accumulations [16].

Furthermore, two markers were used to detect the presence of phosphorylated tau during FC development: CP13 and PHF-1. Granular CP13 immunoreactivity was seen in layer I in a few of the youngest cases in both groups (41 wk NTD and 33 wk DS) (Fig. 8I–L). Although a narrow band of CP13 staining was restricted to layer I in NTD (41 wk), dense and more extensive staining was observed throughout layer I in DS (33 wk) (Fig. 8J, K). CP13-ir cells were not seen in any cortical layer in either group. PHF-1 immunoreactivity was absent in both NTD and DS.

Quantitation of APP/Aβ-ir plaque load and number revealed no significant differences between DS and NTD (Mann–Whitney rank sum test, *p* > 0.05) (Fig. 8M, N).

### FC Co-localization of SMI-32 and Calb

**Fig. 9:**
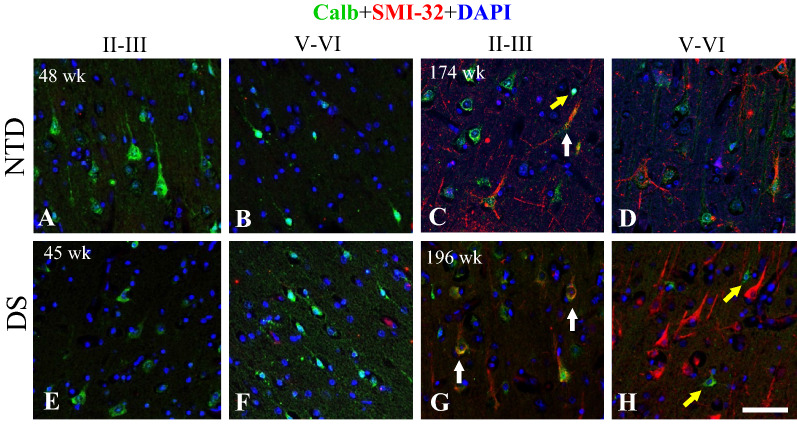
Immunofluorescent images of FC SG and IG layers showing single Calb labeled cells (green) and SMI-32 (red) and dual Calb/SMI32 positive cells (reddish-orange) in 48 (**A**, **B**) and 174 (**C**, **D**) wk NTD and in 45 (**E**, **F**) and 196 (**G**, **H**) postnatal wk-old DS cases. Note that double labeled pyramidal Calb/SMI-32 positive cells (white arrows) were mainly observed in the SG layers in the oldest 174 wk NTD (**C**) and 196 wk DS (**G**) cases, but not in the youngest cases. Small single Calb-ir positive cells (yellow arrows) were seen in SG and IG layers in 174 wk NTD (**C**) and 196 wk DS (**H**), respectively. Blue nuclei were stained with DAPI. Scale bar = 50 µm

To determine whether the long projection neurons labeled with SMI-32 contain the inhibitory marker Calb, we performed dual immunofluorescence. This staining revealed the co-localization of SMI-32 and Calb in pyramidal neurons in SG layer III in the oldest cases in both groups, while in the IG and SG layers, single Calb positive interneurons were observed intermingled with the SMI-32-ir pyramidal neurons in NTD and DS (Fig. 9C, D, G, H). By contrast, only single pyramidal and fusiform Calb, but not SMI-32 labeled cells were found in SG and IG layers in each group in the youngest cases (Fig. 9A, B, E, F).

### Cell counts and demographic correlations

**Fig. 10:**
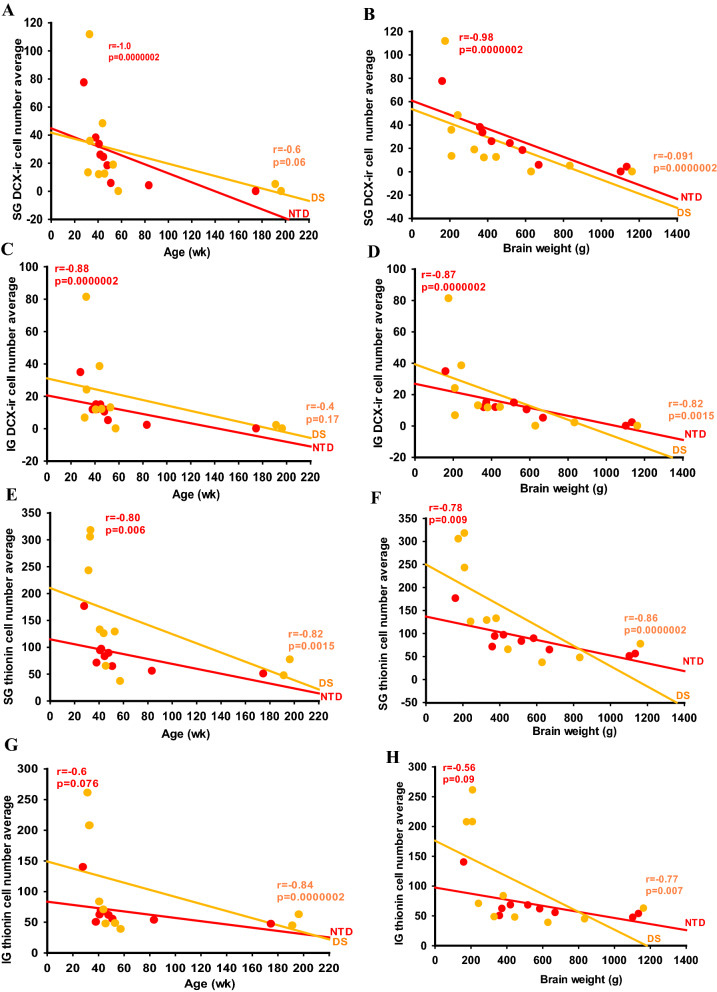
Linear regressions depicting strong negative correlations between DCX cell counts, age and brain weight in SG (**A**, **B**) and IG layers (**C**, **D**) in NTD compared to DS, while thionin counts revealed strong correlations with age and brain weight in SG (**E**, **F**) and IG (**G**, **H**) in DS compared to NTD (Spearman’s rank, FDR: NTD *alpha* < 0.02 and DS *alpha* < 0.007)

**Fig. 11:**
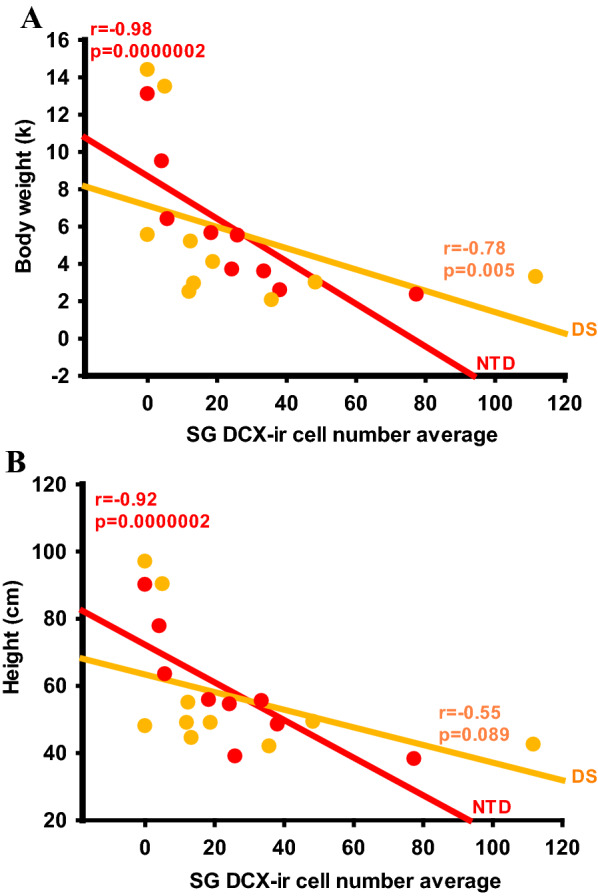
Linear regressions depicting strong negative correlations between SG DCX-ir cell number in the FC and body weight (**A**) and height (**B**) in the NTD group compared to DS (Spearman’s rank, FDR: NTD *alpha* < 0.02 and DS *alpha* < 0.007)

**Fig. 12:**
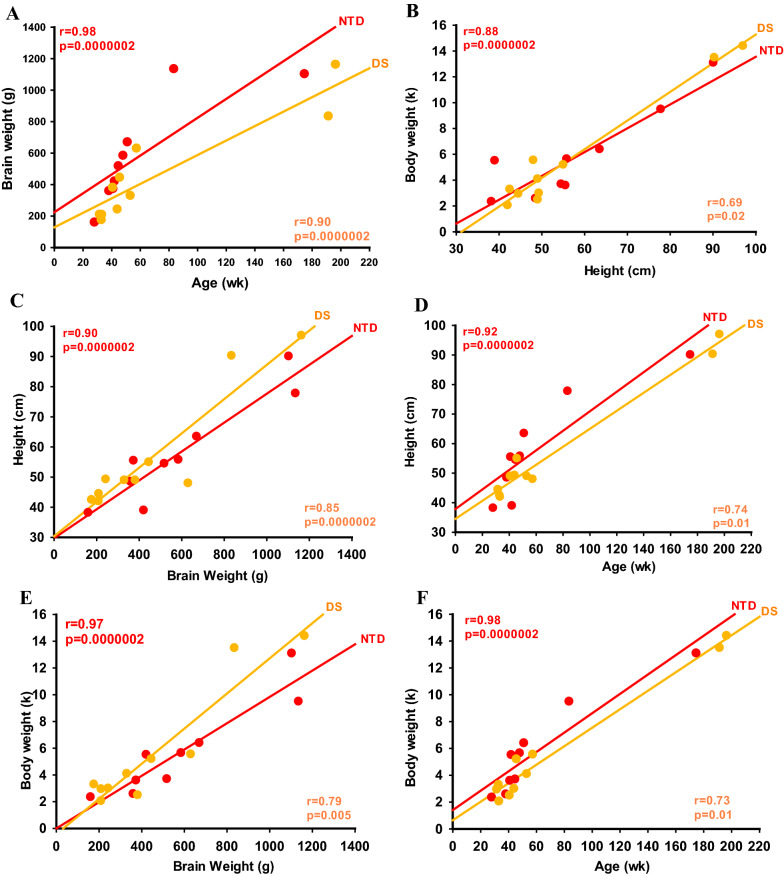
Linear regressions showing stronger positive correlations between brain weight and age (**A**), body weight and height (**B**), height and brain weight (**C**), height and age (**D**), and body weight and brain weight (**E**) and body weight and age (**F**) in NTD compared to DS (Spearman’s rank, *p* < 0.05)

**Fig. 13:**
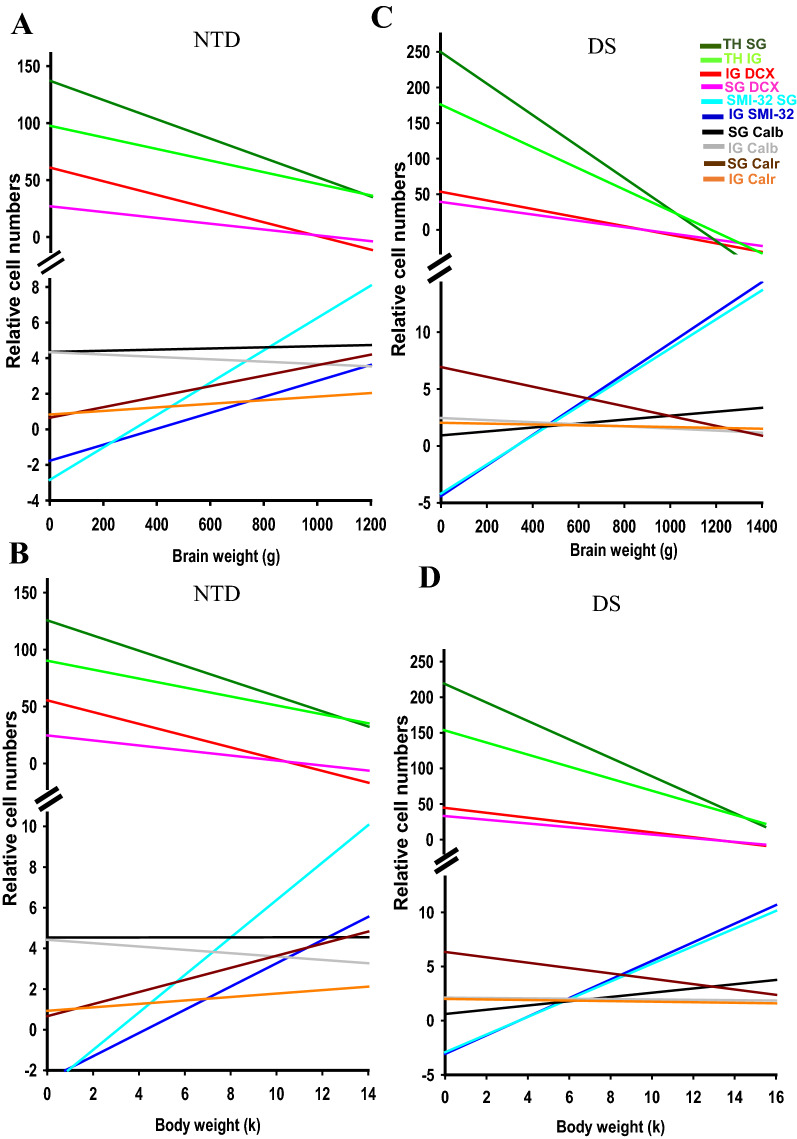
Summary graphs showing the postnatal progression of thionin, DCX, SMI-32, Calb and Calr reactive cell types in relation to brain and body weight in NTD (**A**, **B**) and DS (**C**, **D**). Note the parallel development in the number of different cell types in relation to brain and body weight during growth in NTD (**A**, **B**) and DS (**C**, **D**). Take note that SG (dark green line) and IG (lime green line) thionin cell counts were greater in the youngest DS (**C**, **D**) compared to NTD (**A**, **B**), which decreased with brain and body weight in both groups. Note also that SG Calr (dark brown line) cell numbers were higher in the youngest DS (**C**, **D**) than NTD (**A**, **B**) cases, which decreased with body and brain weight in the former (**C**, **D**), but increased in the latter group (**A**, **B**)

To determine whether there were differential associations between neurogenesis and neuronal differentiation during FC postnatal growth in DS and NTD, correlations between neuronal counts for each cellular marker and demographic variables were performed and only the strongest are reported (Figs. 10, 11, 12, 13; Tables 4, 5, 6, 7).

**Table 4:** Summary of the significant normalized and non-normalized thionin (TH), DCX, SMI-32, Calb and Calr cell count correlations in NTD

NTD	IG TH	SG DCX	IG DCX	IG SMI	IG DCX/IG TH	IG SMI/IG TH	SG Calb/SG TH	SG Calr/SG TH
SG TH	0.90^a^ 2 × 10^−7b^	0.80 0.006	0.88 2 × 10^−7^	− 0.82 0.004	ns	ns	ns	ns
IG TH	–	ns	0.83 0.002	ns	ns	ns	ns	ns
SG DCX	ns	–	0.88 2 × 10^−7^	− 0.90 2 × 10^−7^	ns	ns	ns	ns
IG DCX	ns	ns	–	− 0.75 0.016	ns	ns	ns	ns
SG DCX/SG TH	ns	ns	ns	ns	0.950 2 × 10^−7^	− 0.83 0.002	− 0.83 0.002	− 078 0.009
IG DCX/IG TH	ns	ns	ns	ns	–	− 0.83 0.002	− 0.88 2 × 10^−7^	− 0.86 2 × 10^−7^
IG SMI/IG TH	ns	ns	ns	ns	ns	–	0.83 0.002	
SG Calb/SG TH	ns	ns	ns	ns	ns	ns	–	0.78 0.009

ns, not significant

^a^Spearman’s rank correlation coefficient (r)

^b^FDR: alpha < 0.02

**Table 5:** Summary of significant normalized and non-normalized thionin (TH) DCX, SMI-32, Calb and Calr cell count correlations in DS

DS	IG TH	IG DCX	IG SMI	IG Calr	IG DCX/IG TH	IG SMI/IG TH	IG Calr/IG TH
SG TH	0.93^a^ 2 × 10^−7b^	ns	ns	ns	ns	ns	ns
SG DCX	ns	0.96 2 × 10^−7^	ns	ns	ns	ns	ns
SG SMI	ns	ns	1.00 2 × 10^−7^	ns	ns	ns	ns
SG Calr	ns	ns	ns	0.90 2 × 10^−7^	ns	ns	ns
SG DCX/SG TH	ns	ns	ns	ns	0.95 2 × 10^−7^	ns	ns
SG SMI/SG TH	ns	ns	ns	ns	ns	1.0 2 × 10^−7^	ns
SG Calr/SG TH	ns	ns	ns	ns	ns	ns	0.82 0.00150

ns, not significant

^a^Spearman’s rank correlation coefficient (r)

^b^FDR: alpha < 0.006

**Table 6:** Summary of significant normalized and non-normalized thionin (TH) DCX, SMI-32, Calb and Calr cell counts, age, brain and body weight and height correlations in DS and NTD

NTD	Age	Brain weight	Body weight	Height	DS	Age	Brain weight	Body weight	Height
SG TH	− 0.80^a^ 0.006^b^	− 0.78 0.009	− 0.75 0.016	− 0.85 0.0004		− 0.82 0.0015	− 0.86 2 × 10^−7^	ns	ns
IG TH	ns	ns	ns	ns		− 0.84 2 × 10^−7^	− 0.77 0.007	ns	ns
SG DCX	− 1.00 2 × 10^−7^	− 0.98 2 × 10^−7^	− 0.98 2 × 10^−7^	− 0.92 2 × 10^−7^		ns	− 0.91 2 × 10^−7^	− 0.78 0.005	ns
IG DCX	− 0.88 2 × 10^−7^	− 0.87 2 × 10^−7^	− 0.90 2 × 10^−7^	− 0.85 0.0004		ns	− 0.82 0.0015	ns	ns
IG SMI-32	0.91 2 × 10^−7^	0.88 2 × 10^−7^	0.83 0.002	0.83 0.002		ns	ns	ns	ns
SG DCX/SG TH	− 0.97 2 × 10^−7^	− 0.95 2 × 10^−7^	− 0.98 2 × 10^−7^	− 0.85 0.0004		ns	ns	ns	ns
IG DCX/IG TH	− 0.97 2 × 10^−7^	− 0.95 2 × 10^−7^	− 0.98 2 × 10^−7^	− 0.85 0.0004		ns	ns	ns	ns
IG SMI-32/IG TH	0.91 2 × 10^−7^	0.89 2 × 10^−7^	0.83 0.002	0.83 0.002		ns	ns	ns	ns
SG Calb/SG TH	0.88 2 × 10^−7^	0.92 2 × 10^−7^	0.87 2 × 10^−7^	0.73 0.02		ns	ns	ns	ns
SG Calr/SG TH	0.76 0.016	ns	0.82 0.004	ns		ns	ns	ns	ns

ns, not significant

^a^Spearman’s rank correlation coefficient (r)

^b^FDR: NTD alpha < 0.02 and DS alpha < 0.007

**Table 7:** Correlations between age, brain and body weight and height in DS and NTD

	Age	Brain weight	Body weight	Height
*NTD*				
Age	–	0.98^a^ 2 × 10^−7b^	0.98 2 × 10^−7^	0.92 2 × 10^−7^
Brain weight	–	–	0.97 2 × 10^−7^	0.90 2 × 10^−7^
Body weight	–	–	–	0.88 2 × 10^−7^
Height	–	–	–	–
*DS*				
Age	–	0.90 2 × 10^−7^	0.73 0.01	0.74 0.01
Brain weight	–	–	0.79 0.005	0.85 2 × 10^−7^
Body weight	–	–	–	0.69 0.02
Height	–	–	–	–

^a^Spearman’s rank correlation coefficient (r)

^b^*p* value

In NTD, SG thionin counts positively correlated with SG (Spearman’s rank, *r* = 0.80, *p* = 0.006) and IG DCX-ir counts (Spearman’s rank, *r* = 0.88, *p* = 0.0000002), and negatively with IG SMI-32 values (Spearman’s rank, *r* = − 0.82, *p* = 0.004), while IG thionin counts showed a positive correlation with IG DCX (Spearman’s rank, *r* = 0.83, *p* = 0.002) (Table 4). IG SMI-32-ir counts displayed a strong negative correlation with SG DCX (Spearman’s rank, *r* = − 0.90, *p* = 0.0000002), but weaker with IG DCX in NTD (Spearman’s rank, *r* = − 0.75, *p* = 0.016) (Table 4). Thionin and DCX-ir cell counts in SG and IG showed a strong positive correlation with each other in NTD (Spearman’s rank, thionin *r* = 0.90, *p* = 0.0000002; DCX *r* = 0.88, *p* = 0.0000002) and in DS (Spearman’s rank, thionin *r* = 0.93, *p* = 0.0000002; DCX *r* = 0.96, *p* = 0.0000002) (Tables 4 and 5). SMI-32 and Calr-ir cell counts in SG and IG were positively correlated with each other in DS (Spearman’s rank, SMI-32 *r* = 1.0, *p* = 0.0000002; Calr *r* = 0.90, *p* = 0.0000002) (Table 5), but not in NTD (Spearman’s rank, SMI-32 *r* = 0.67, *p* = 0.043; Calr *r* = 0.66, *p* = 0.043). Moreover, normalized IG DCX cell counts showed a strong negative correlation with normalized SG Calb and Calr cell counts in NTD (Table 4). In DS, normalized DCX, SMI-32 and Calr cell count correlations were similar to non-normalized counts (Table 5).

Age and brain weight displayed strong positive correlations with IG SMI-32 cell counts (Spearman’s rank, age *r* = 0.91, *p* = 0.0000002; weight: *r* = 0.88 *p* = 0.0000002) (Table 6) but correlated negatively with SG (Spearman’s rank, age: *r* = − 1.0, *p* = 0.0000002; brain weight, *r* = − 0.98, *p* = 0.0000002) (Fig. 10A, B; Table 6) and IG DCX cell counts (Spearman’s rank, age, *r* = − 0.88, *p* = 0.0000002; brain weight *r* = − 0.87, *p* = 0.0000002) (Fig. 10C, D; Table 6), and weakly with SG thionin cell counts (Spearman’s rank, age: *r* = − 0.80, *p* = 0.0062; brain weight: *r* = − 0.78, *p* = 0.009) in NTD (Fig. 10E, F; Table 6). In the DS group, age and brain weight correlated negatively with SG thionin neuronal counts (Spearman’s rank: age *r* = − 0.82, *p* = 0.0015; brain weight *r* = − 0.86, *p* = 0.0000002), whereas age was highly negatively correlated with IG thionin cell number (Spearman’s rank, age *r* = − 0.84, *p* = 0.0000002; brain weight *r* = − 0.77, *p* = 0.007) (Fig. 10G, H; Table 6). However, only brain weight showed a strong negative correlation with SG DCX values (Spearman’s rank, brain weight *r* = − 0.91, *p* = 0.0000002; age *r* = − 0.82, *p* = 0.0015) in DS (Table 6). Finally, body weight and height displayed robust negative associations with SG (Spearman’s rank, body weight *r* = − 0.98, *p* = 0.0000002; height *r* = − 0.92, *p* = 0.0000002) (Fig. 11A, B; Table 6) and IG DCX-ir cell counts (Spearman’s rank, body weight *r* = − 0.90, *p* = 0.0000002; height *r* = − 0.85, *p* = 0.0004) (Table 6) in the NTD group. However, only body weight and DCX cell counts were significantly negatively correlated within SG (Spearman’s rank, *r* = − 0.78, *p* = 0.005), but not IG in DS (Table 6). In NTD, age, brain and body weight, and height showed negative correlations with normalized IG and SG DCX cell counts and positively correlated with normalized IG SMI-32 values (Table 6). Additionally, age, brain and body weight, and height correlated positively with normalized Calb cell numbers in the SG layers, while only age and body weight correlated with normalized SG Calr counts in NTD (Table 6). In contrast, there were no significant correlations between age, brain and body weight, or height with any normalized cell counts in DS (Table 6).

Brain weight and age showed a stronger positive association in NTD (Spearman’s rank, *r* = 0.98, *p* = 0.0000002) compared to DS (Spearman’s rank, *r* = 0.90, *p* = 0.0000002) (Fig. 12A; Table 7). The association between body weight and height was stronger in NTD (Spearman’s rank, *r* = 0.88, *p* = 0.0000002) compared to DS (Spearman’s rank, *r* = 0.69, *p* = 0.02) (Fig. 12B; Table 7). In addition, height and body weight displayed a stronger correlation with brain weight and age in NTD (Spearman’s rank, *r* ≥ 0.90, *p* = 0.0000002) than in DS (Spearman’s rank, *r* = 0.73–0.85, *p* ≤ 0.02) (Fig. 12C–F; Table 7).

## Discussion

The layered cerebral cortex results from a series of complex harmonized events during prenatal and postnatal development. Consequently, projection and local neurons yield intricate neuronal architecture and connectivity patterns that underlie the ability of humans to perform complex behavioral functions. Disturbances in cortical development lead to changes in motor, sensory, behavioral, and cognitive function in newborns, infants, children, and adolescents. DS is characterized by an array of cognitive difficulties starting early in life [27], which are attributed to deficits in cortical development including cell proliferation and migration, apoptosis, neurogenesis, synaptogenesis and gliogenesis [28, 32, 51], particularly in the FC. Although many of these processes have been studied during the fetal and prenatal stages of brain development in DS [5, 14, 28, 32, 42, 49, 69, 72, 86], there is a general lack of information on early postnatal brain maturation in DS. Here, we evaluated postnatal cellular changes in the FC of infants and children with DS to gain greater insight into the effect of trisomy 21 upon cortical development that may provide clues to therapeutic targets to prevent or slow cognitive disability in children with DS.

### Postnatal FC lamination and cytoarchitecture in DS

In this study, thionin histochemistry was used to investigate the postnatal maturation of different neuronal cell types within the layers of the FC in infants and children with DS compared to NTD. We found that lamination and cellular distribution in the FC was well-organized and distinguishable in all NTD cases examined, as previously described [61]. In contrast, in DS, cell layers were less well-defined and displayed poor cellular organization [6]. In both groups, layers II and IV were distinct in premature infants (28 wk NTD; 31 wk DS), while in term newborns, layers II and IV were clearly observable in DS, but not in NTD, perhaps reflecting an abnormal distribution of the neurons and/or a greater delay in cell maturation in cortical layers II and IV [91]. Comparatively, cortical layers were less well-developed and more densely packed at all ages in DS. However, differences in cell number were not seen between the SG and IG layers between groups. In all cases the SG layers displayed a greater cellular density than the IG in both groups. In addition, we observed a delay in cellular maturation in the different cortical layers in DS compared to NTD cases. In fact, the thionin-stained pyramidal-shaped cells were first observed in layer V in a 28 wk-premature NTD infant, compared to at 44 wk in DS. Furthermore, in the oldest NTD subjects, cells in the SG and IG layers had a more classic pyramidal shape and were better organized than in the oldest DS. These findings support a previous study showing altered cortical lamination and reduced cellular proliferation in prenatal human cases with DS [13]. Together these findings indicate a spatiotemporal delay in FC organization and neuronal maturation, likely affecting cortical function early in DS.

### Postnatal FC proliferation and neurogenesis in DS

During both prenatal and postnatal development, cells proliferate, migrate and differentiate within the FC [73, 90]. These coordinated events are critical for the normal neuronal FC development. Cell proliferation is revealed by the nuclear human Ki-67 protein [77], which is seen during the active phases of the cell cycle but is absent in resting cells [20, 77]. In contrast to a previous report [68], we did not detect Ki-67 immunoreactivity in the postnatal FC in NTD or DS subjects. Similarly, Ki-67 positive cells were not found in the postnatal hippocampus in NTD or DS [60]. The discrepancy between these may be related to the observation that proliferation markers, including Ki-67, show a steady and rapid postmortem decline at least in rats [79]. Whether this occurs in the human brain remains to be investigated.

The microtubule-associated protein DCX, a marker of neurogenesis that affects microtubule stabilization and cellular dynamics [4], is expressed in neuroblasts and migrating neurons during embryonic and postnatal development of the central and peripheral nervous systems [26]. Here, DCX immunoreactivity was observed in the youngest NTD and DS infants (28 wk NTD; 31 wk DS). Although DCX-ir cells were observed in SG layer II, reactivity decreased with age in both NTD and DS. Similar findings have been reported in the human cortex where DCX was highly expressed soon after birth and declined dramatically in the first two years of life [24, 68]. In the postnatal rhesus monkey FC, DCX-ir cells were seen in layer II at 12 days and again at 1 month of age [24]. Although we found that an increase in age was associated with a decrease in DCX in both groups, positive cells decreased more rapidly in the postnatal DS compared to NTD cases. Furthermore, we demonstrated that the number of DCX-ir cells in the SG and IG layers correlated negatively with age, brain weight and height in NTD, but not in DS. These findings suggest that cortical neurogenesis is a time-dependent event strongly associated with body/brain growth that is downregulated early during postnatal development likely affecting connectivity, physiology and function of the FC in DS [9]. We did not detect differences between Ki-67 immunoreactivity compared to a decrease in DCX positive cells in both groups, suggesting that cell proliferation ceases prior to neurogenesis in the postnatal FC in both DS and NTD.

### Postnatal FC NHF reactivity in DS

Neurofilaments are cytoskeletal polymers that play an important role in the maintenance of large neurons with highly myelinated processes [67]. Here, we used the SMI-32 antibody that recognizes non-phosphorylated epitopes of the heavy-weight neurofilament proteins that are preferentially expressed in the dendrites and soma of mature pyramidal neurons [18, 36, 55, 66, 89]. We observed that most SMI-32-labeled large pyramidal neurons were located in the IG layers (V-VI) as early as 28 wk in NTD but much later (196 wk) in DS. SMI-32 positive pyramidal cells in layer III were observed at age 41 wk in NTD, but not until age 196 wk in DS. Our results support the concept that maturation of layer V pyramidal neurons precedes their maturation in layer III in NTD [61], compared to delayed maturation of these large projection neurons in layers III and V in the FC in DS. The lack of NHF in the pyramidal cells in DS (present findings) likely contributes to the smaller dendritic arborization and fewer synapses reported in pyramidal neurons in the postnatal DS cortex [32]. We observed intense SMI-32 immunostaining of large pyramidal neurons in layer V in the oldest cases from both groups, supporting the concept that neurofilaments increase with cell size [89]. Interestingly, in the monkey cortex, SMI-32 is differentially expressed in subpopulations of pyramidal cells in layer V, with the highest expression seen in pyramidal cells that give rise to corticocortical projections [10]. Therefore, it is likely that the SMI-32 positive pyramidal cells found here belong to a population of corticocortical projection neurons. However, further studies are needed to investigate the developmental effects on pyramidal neurons and the influence on FC corticocortical connectivity during development in DS. Moreover, SMI-32 positive pyramidal cell numbers in layer V showed a strong positive correlation with age, brain and body weight, and height in NTD. These findings suggest that brain maturation and body growth are harmonized during postnatal development in NTD, but not in DS. Overall, maturation of FC pyramidal neurons is delayed in early postnatal development in DS and may underlie the impairment of executive function seen during childhood and adolescence in DS.

### Postnatal FC CBP in DS

The CBP markers, Calb, Calr, and Parv, are present in GABAergic inhibitory cells that employ γ-aminobutyric acid [35]. During fetal human cortical development CBP-containing cells play a role in the establishment of transitory neuronal circuits, which are essential for the formation of mature neuronal circuits [88] through the mediation of cortical wiring, plasticity, and inhibitory neurotransmission [43]. In DS, it has been hypothesized that GABAergic dysfunction impairs synaptic plasticity, learning and memory by altering the optimal balance between excitatory/inhibitory synapses [15].

In the present study, we detected Calb-ir profiles in the FC at all ages in both NTD and DS, similar to that reported in the hippocampus [60]. The presence of Calb-ir cells at birth in the FC is similar to that reported in human postnatal entorhinal and visual cortex [29, 47] as well as the rodent neocortex [1]. These data suggest that Calb FC circuits develop early in life and continue throughout the postnatal period. While the first Calb-ir cells in the FC were observed at 28 wk in a premature NTD infant [88], only neuropil staining was detected in premature infants with DS (present findings). Calb-ir positive neurons in SG and IG layers were seen at 41 wk of age in NTD and at 44 wk in infants with DS. Comparatively, these Calb immunolabeled cells are smaller with less distinctive processes in DS, even in the oldest cases, indicative of a developmental delay of these interneurons early in the postnatal DS cortex. Moreover, we found that Calb-ir cell numbers were significantly lower in both the SG and IG layers in DS compared to NTD. Normalized Calb-ir cell counts correlated positively with age, brain and body weight, and height in NTD, but not in DS. Similar to our postnatal findings, Calb-ir non-pyramidal neurons were greatly reduced in the FC in elderly DS [41]. However, it is unknown whether Calb-ir cell numbers in the FC are consistently lower throughout life in DS.

Unlike Calb, we detected Calr-ir cells as early as 32 wk in DS compared to 44 wk in NTD. These cells were mainly observed in layers II/III in both groups, comparable to that seen in the human postnatal entorhinal cortex [29] in NTD subjects. Calr-ir cells were more numerous in both SG and IG layers in DS compared to NTD, similar to our previous findings in the postnatal hippocampus in DS and NTD [60]. However, a previous study using human-derived euploid induced pluripotent stem cells (iPSCs) showed that, in DS, there were significantly fewer Calr-ir interneurons compared to non-DS models [37]. This iPSC study suggested that DS GABAergic interneurons, including Calr positive cells, exhibit decreased migration in vitro during development [37]. These discrepancies in cortical Calr numbers may be related to the comparison between examining human DS tissue in the present study and the forced induction of neuronal phenotypes in in vitro models of DS. Despite higher numbers of cortical Calr positive cells exhibited in postnatal DS cases, we found strong positive associations between normalized Calr positive cell counts in the SG and IG with age, brain and body weight, and height in the NTD group, but not in DS.

Parv-ir cells in the FC were first observed in the IG layer at 196 wk in DS, but not at 174 wk in NTD. Conversely, no Parv-ir cells were detected in the postnatal hippocampus at any age in either DS or NTD [60]. Moreover, Parv-ir cells were found to be absent at birth in the human entorhinal and visual cortex in neurotypical babies, but present later during the postnatal period [29, 47]. Even though a reduction in the number of Parv-ir interneurons was reported in the cerebral cortex in elderly people with DS [41], further investigation on FC neuronal Parv differentiation in the postnatal period is required. Taken together, these data support the hypothesis that GABAergic neuronal dysfunction plays a role in cortical circuit development, leading to intellectual disability early in life in DS, which extends to adulthood [94]. While numerous studies have demonstrated that GABAergic drugs rescue behavioral deficits in animal models of DS [21, 53, 74], clinical trials targeting GABAergic signaling have failed to meet their primary cognitive endpoints in patients with DS [44]. More research is needed to better understand the role of GABAergic neuronal dysfunction as a therapeutic target to treat cognitive impairment in DS.

### Postnatal FC APP and Aβ_1–42_ in DS

APP is a transmembrane protein highly expressed in the FC [65] and plays a role in cellular growth, cellular differentiation, cell–cell communication and synaptic plasticity throughout life [63, 65]. In pathological conditions, the proteolysis of APP generates Aβ peptides, which accumulate to form Aβ plaques, a hallmark of Alzheimer’s disease (AD) [65]. In DS, the gene for *APP* is triplicated, due to the presence of an extra full or partial chromosome 21, leading to increased production of toxic Aβ_1–42_ [8] and amyloid plaque deposition beginning as early as the late teens [17, 45, 85]. We detected diffuse plaque-like APP/Aβ-ir accumulations scattered throughout all layers of the FC in both DS and NTD cases. However, no Aβ_1–42_ immunoreactivity was detected in any case examined. Similar findings have been reported in the postnatal hippocampus in DS and NTD [60]. In DS, Aβ soluble species, which precede plaque deposition, have been reported as early as 21 gestational wk [30, 32]. A prior study reported no amyloid plaque pathology at 0.01, 1.6 and 3 months of age in the frontal and temporal cortex or brainstem in DS [17]. High expression of certain isoforms of APP occurs in cortex at birth and at postnatal day 10 in rats, suggesting a role in the postnatal regulation of cellular growth and synaptogenesis [3]. Interestingly APP mRNA and protein levels increased two-fold over the course of neuronal differentiation in a DS isogenic human model [67]. Since we did not detect Aβ_1–42_ immunostaining in the FC, we suggest that the diffuse plaque-like accumulations reported here contain non-pathological APP or derivatives of this protein in both NTD and DS. Although several studies have demonstrated that overexpression of cortical APP, S100B, and OLIG2 impair proliferation/neurogenesis in the fetal DS brain [49, 50], the effect that the overexpression of *APP* alone or in conjunction with other genes located on chromosome 21 (e.g., S100B, DYRK1A*,* RCAN1*,* OLIG1/2, SOD1) has upon FC postnatal maturation requires further investigation [7, 59].

### Postnatal FC Tau in DS

Along with Aβ plaques, the other classic pathological hallmark of AD, NFTs, are composed of phosphorylated tau [56]. The normal biological function of tau is the assembly and stabilization of microtubules to regulate neuritic growth [39]. Hyper-phosphorylation of tau results in the loss of physiological function and its aggregation in select brain regions, which contributes to learning and memory impairments reported in various tauopathies [54, 56]. NFTs develop by the forties and are linked to the cognitive impairment in DS [34, 52, 70, 71]. The shortest tau isoform is highly expressed throughout fetal development, but particularly during midgestation [39]. The normal biological function of tau involves the assembly and stabilization of microtubules to regulate neuritic growth [39]. Phosphorylation of fetal tau occurs in the distal portion of growing axons, which is downregulated after 35 wk gestation [39]. Different abnormal tau phosphorylation events during fetal development (14–28 wk gestation) have been investigated using tau epitope-specific antibodies revealing tau positive white matter tracts (e.g., cerebellar peduncles and internal capsule) [56, 57, 80] suggesting early axonal transport defects, a common feature in tauopathies [80]. Interestingly, we reported a band of phosphorylated CP13 (Ser202) and PHF-1 (Ser396) tau immunoreactivity located between the external granular (or germinal) and molecular layers of the cerebellum during early postnatal development [62]. Our findings of phosphorylated tau CP13 in postnatal FC layer I, but not PHF-1, in younger NTD and DS cases, suggests the expression of an early non-pathological form of tau [39, 93]. In a previous study, we did not detect CP13 immunostaining during postnatal development of the hippocampus in either DS or NTD [60]. A recent imaging study using the tau tracers 3H-THK5117 and 3H-MK6240 demonstrated binding in the DS fetal cortex, but not in control cases [46]. Although the functional significance of cortical tau remains unclear in DS, it may affect synaptic formation, neuronal sprouting or pruning during development [34].

### Relationship between FC neuronal profiles and biometrics

Brain growth during the prenatal and postnatal periods is a reflection of cell proliferation and migration, as well as neuronal, soma, axonal, and dendritic growth, synaptogenesis, glial cell proliferation, and myelination. Although we did not see significant differences in brain weight and age between groups, correlations were weaker in DS than in NTD, where brain growth is slower in DS than in NTD [76]. Body height and weight were strongly associated with age and brain weight in NTD, but weakly in DS. Brain weight and age correlated more strongly with thionin, DCX, and SMI-32 cell counts in NTD compared to DS. Similarly, we previously reported a strong negative correlation between dentate gyrus DCX cell counts and brain weight in DS compared to NTD [60]. Body height and weight were negatively correlated with cell counts for the neuronal migration marker DCX in NTD, but not in DS. Altogether these data indicate that neurogenesis at postnatal ages follows a coordinated timeline with brain/body growth that is impaired during postnatal FC development, similar to the hippocampus [60] in DS.

### Study limitations

There are limitations to our study. In this regard, varying degrees of functional impairment exist in individuals with DS suggesting the existence of differences in fetal/early neuronal developmental abnormalities between individuals/infants with this syndrome. Variable postmortem intervals across cases may affect tissue quality. Since there were limited cases examined at each age/stage, the present findings should be interpreted with caution and confirmed in a larger cohort. This caveat is due to an inadequate number of brain banks that collect human DS and NTD postmortem tissue at all ages. To assist in this endeavor, we established the Down Syndrome BioBank Consortium (http://devdownsbio.wpengine.com/families-donors/brain-donation-registry/) funded by the BrightFocus Foundation.

## Summary and conclusions

In sum, the postnatal FC displays a spatiotemporal delay in lamination, poor cellular organization and delayed neuronal differentiation in DS. DCX-ir cells declined with age in both NTD and DS, however they decreased more rapidly in DS. SMI-32-ir cells were detected in NTD much earlier than DS, in which SMI-32 was only detected at 196 wk. In NTD, the maturation of SMI-32-ir pyramidal neurons in layer V preceded layer III, however DS pyramidal neuronal maturation was simultaneously delayed in layers III and V. Calb-ir cell numbers were significantly higher in NTD. Calb-ir neurons were first seen at 41 wk NTD compared to 44 wk in DS, and DS Calb-ir interneurons displayed morphological defects. Calr-ir cells were observed as early as 32 wk in DS compared to 44 wk in NTD and were more numerous across all cortical layers in DS. Parv immunoreactivity was only detected in the IG layer at 196 wk in DS. DCX-ir, Calb-ir, and Calr-ir cell numbers were positively correlated with age, brain/body weight, and height in NTD, but not in DS. APP/Aβ-ir diffuse accumulations were detected in all layers of the FC in both DS and NTD cases, however Aβ_1–42_ plaques/accumulations were not detected in any sample. Phosphorylated tau CP13 was seen in layer I, but not PHF-1, at 41 wk and 33 wk in NTD and DS, respectively. These findings suggest that trisomy of chromosome 21 affects spatiotemporal postnatal development of FC lamination, neuronal migration/neurogenesis, differentiation and phenotypic maturation of projection pyramidal cells and interneurons (see Fig. 13), which contribute to the impaired cognition seen in this developmental disorder. The effect(s) that alterations to the neuronal substrate of the FC has upon behavior in DS remains an intriguing area of research. Functionally, children with DS show impairments in episodic and executive function, working memory and attention [92] mediated, in part, by the FC. It is well established that individuals with DS display a shrunken FC [62] and that preterm and term infants and children with this disorder exhibit a reduction in frontal lobe functional connectivity [38, 92] suggesting that impairment of cortical connectivity is a core mechanism(s) underlying cognitive impairment(s) for people with DS. In addition, phospho-tau CP13, but not PHF-1, was seen in FC postnatal layer I in both young NTD and DS cases. Although the precise functional significance that postnatal tau has upon neuronal organization remains unknown, it may also affect cognitive and attentional behavior(s) throughout the lifespan of individuals with DS [34].

## Data Availability

The raw data that support the findings of this study are available from the corresponding author upon reasonable request.
